# Transactions of the New York Pathological Society

**Published:** 1878-01

**Authors:** 


*Transactions of the New York Pathological Society.* Volume II. Based on the proceedings of the year 1875 and largely supplemented from the records of 1844 to 1877. Edited by JOHN C. PETERS, M. D. New York: Printed for the Society by Wm. Wood & Co. 1877. Buffalo: H. H. Otis.

In noticing the first volume we expressed the hope that other volumes would follow at short intervals and we are glad that the society have so soon given the second of its publications to the profession, but we are sorry that the good work is likely to be interrupted from lack of funds. The society is so rich in pathological material of great practical importance that it is to be hoped that some way may be found to publish still more of its proceedings.

The present volume is devoted to diseases of the intestines and diseases of the liver. We have not the space to name the many rare and interesting cases presented, but can recommend it as worthy of close attention. As the edition of each volume is small it would be well for all who wish to purchase to do so at once else they may be unable to procure a copy. C.

